# Spatiotemporal Changes Posttreatment in Peripheral Arterial Disease

**DOI:** 10.1155/2015/124023

**Published:** 2015-12-03

**Authors:** Sara A. Myers, Neil B. Huben, Jennifer M. Yentes, John D. McCamley, Elizabeth R. Lyden, Iraklis I. Pipinos, Jason M. Johanning

**Affiliations:** ^1^Center for Research in Human Movement Variability, School of Health, Physical Education, and Recreation, University of Nebraska Omaha, 6160 University Drive South, Omaha, NE 68182-0860, USA; ^2^College of Public Health, Department of Biostatistics, University of Nebraska Medical Center, 984355 Nebraska Medical Center, Omaha, NE 68198, USA; ^3^Department of Surgery, Omaha Veterans Affairs Medical Center, 4101 Woolworth Avenue (121), Omaha, NE 68105, USA; ^4^College of Medicine, Department of Surgery, University of Nebraska Medical Center, 983280 Nebraska Medical Center, Omaha, NE 68198, USA

## Abstract

Accumulating evidence suggests revascularization of peripheral arterial disease (PAD) limbs results in limited improvement in functional gait parameters, suggesting underlying locomotor system pathology. Spatial and temporal (ST) gait parameters are well studied in patients with PAD at baseline and are abnormal when compared to controls. The purpose of this study was to systematically review and critically analyze the available data on ST gait parameters before and after interventions. A full review of literature was conducted and articles were included which examined ST gait parameters before and after intervention (revascularization and exercise). Thirty-three intervention articles were identified based on 154 articles that evaluated ST gait parameters in PAD. Four articles fully assessed ST gait parameters before and after intervention and were included in our analysis. The systematic review of the literature revealed a limited number of studies assessing ST gait parameters. Of those found, results demonstrated the absence of improvement in gait parameters due to either exercise or surgical intervention. Our study demonstrates significant lack of research examining the effectiveness of treatments on ST gait parameters in patients with PAD. Based on the four published articles, ST gait parameters failed to significantly improve in patients with PAD following intervention.

## 1. Introduction

Peripheral arterial disease (PAD) is the result of atherosclerosis in the lower extremities, affecting 8 to 12 million people in the United States and up to 30% of older adults [1, 2]. PAD is often accompanied by intermittent claudication that occurs due to ischemic conditions after lower extremity blood flow decreases. Intermittent claudication is defined as pain occurring during physical exertion that ceases after rest. Patients with PAD commonly present with intermittent claudication after walking a short distance and complain of having to cease physical activity to allow for reperfusion [3]. Thus, PAD is a painful ambulatory disorder of the lower extremities that originates during and ultimately halts ambulation. Underlying this ambulatory dysfunction are severe musculoskeletal changes and myopathy [4–6] that may be related to abnormal gait patterns and lead to a loss of function. Thus, PAD results in reduced physical functioning and higher prevalence of falling and is associated with increased cardiovascular morbidity and mortality [7, 8].

Ambulation requires the integration of numerous systems and will reflect the overall burden of disease [9]. Spatial and temporal (ST) gait parameters are commonly used to identify abnormalities in ambulation and to monitor patient progress. ST gait parameters have also been proposed as global markers for health-related risk in older adults [10]. Walking velocity alone has been shown to be an independent predictor of hospitalization and disability [11–13]. Variabilities in step length and stance time have also been associated with increased health problems, functional loss, and decreased physical activity [14]. Consequently, evaluation of ST gait parameters may be used as a surrogate measure to assess overall functional status.

Previous studies of patients with PAD have utilized measurement of ST gait parameters to document the functional loss due to abnormal gait patterns that result from PAD [15–18]. In particular, patients with PAD demonstrate decreased walking velocity, cadence, step length, stride length, time spent in single support, and increased time spent in double support during pain-free conditions, when compared to control subjects [15, 19]. When patients are asked to walk while experiencing claudication pain, further decrements in ST gait parameters have been recorded [20]. With increasing deficits in gait parameters as the disease progresses, there is a need to investigate the efficacy of treatment programs for these patients.

Several treatments are available to address functional loss in PAD. Currently, primary medical treatments for patients with PAD include revascularization, pharmacology, risk factor management (i.e., smoking cessation), and exercise programs that focus on restoring impaired physical functioning. Several meta-analyses have been performed to evaluate the effectiveness of different treatments on improving initial claudication distance (also referred to as pain-free walking distance) and the distance covered during the 6-minute walk test [21–24]. It has been found that pharmacological treatments, pentoxifylline and cilostazol, significantly improve initial claudication distance for patients with PAD up to six months after the initiation of treatment [21, 22]. Supervised treadmill training [23], strength training [25], and high intensity resistance training [26] have been shown to significantly improve 6-minute walking distance while strength training also improved initial claudication distance [27]. These walking distance-based measures provide only a general measure of functional loss [28] and do not provide insight into ambulatory dysfunction associated with PAD. Evaluation of basic ST parameters of gait is a necessary first step to provide such insight. Evaluation of the literature, however, reveals that there has been no systematic review of studies utilizing ST gait parameters to evaluate the effectiveness of treatments on functional improvements.

The purpose of this study was to systematically review and critically analyze available data on ST gait parameters, before and after treatments, and consequently provide an overall evaluation of changes in functional status posttreatment. If sufficient studies have been conducted to evaluate ST gait parameters before and after treatment, this review will shed light on which treatments most effectively lead to functional improvements. If insufficient studies have investigated ST gait parameters before and after treatment, the review will demonstrate the need for future studies of changes in ST gait parameters following treatment of patients with peripheral arterial disease.

## 2. Methods

This review was performed following procedures outlined in previous reviews of treatment outcomes for intermittent claudication patients [29–31].

### 2.1. Literature Search Strategy

The search strategy was developed to identify all articles pertaining to basic ST gait parameters in patients with PAD. Databases searched were PubMed (from 1950), EBSCO (from 1982), CINAHL (from 1982), Cochrane library (from 1900), and UMI Dissertation (from 1963). Targeted searching of frequently cited journals, authors, and article reference lists ensured that all relevant articles were obtained.

For an article to be labeled relevant for review, it had to contain words pertaining to basic ST gait parameters in patients with PAD in the title, abstract, or keywords. Search strings included “walking velocity and peripheral arterial disease,” “cadence and peripheral arterial disease,” “stride length and peripheral arterial disease,” “step width and peripheral arterial disease,” “walking velocity and intermittent claudication,” “cadence and intermittent claudication,” “stride length and intermittent claudication,” and “step width and intermittent claudication.”

### 2.2. Study Selection

The initial search yield was obtained by combining all original articles from online databases. All original articles were available to the authors. Articles not initially available were obtained via interlibrary loan. Articles not pertaining to basic ST gait parameters in patients with PAD were excluded during initial screening of titles and abstracts by the reviewer (author: Neil B. Huben). The full text of the remaining articles was then screened by the reviewer (author: Neil B. Huben). There was no date restriction on articles. Articles were required to be full text in English. The articles containing mean and standard deviation information for the basic ST gait parameters in patients with PAD were further screened for the inclusion of pre- and posttreatment data for walking velocity, cadence, stride length, and step width parameters following treatment by two reviewers (authors: Neil B. Huben and Jennifer M. Yentes).

### 2.3. Quality Assessment

For systematic literature reviews, it is important to conduct a quality assessment to decrease reviewer bias and increase standard of review [32]. In order to conduct a systematic assessment, three reviewers (authors: Jason M. Johanning, Neil B. Huben, and Jennifer M. Yentes) developed a table checklist based on standardized techniques presented in published reviews and literature sources on systematic review principles [32–35].

The quality assessment table developed for the current systematic review, Table 1, was formulated based on the fundamental research aims. Two reviewers (authors: Neil B. Huben and Jennifer M. Yentes) analyzed and scored the quality of each article independently. Differences in scores after independent analysis were discussed by the initial reviewers (authors: Neil B. Huben and Jennifer M. Yentes), plus one additional third-party reviewer (author: Sara A. Myers).

### 2.4. Data Extraction

A customized data extraction document which formed the questions for Table 1 was created and agreed upon by two authors (authors: Jason M. Johanning and Neil B. Huben) to ensure extraction reliability. The major data extraction categories included type of treatment, ankle-brachial index, walking velocity, cadence, stride length, and step width. Data extraction was performed initially by one reviewer (author: Neil B. Huben) and then checked by two additional reviewers (authors: Jason M. Johanning and Jennifer M. Yentes).

### 2.5. Reliability of the Reviewing Process

The quality of the selected articles was analyzed independently by two researchers (authors: Neil B. Huben and Jennifer M. Yentes). To verify the reliability of quality assessment, two reviewers (authors: Neil B. Huben and Jennifer M. Yentes) independently assessed a single, randomized pilot article and their results were checked by a third reviewer (author: Sara A. Myers). To verify the reliability of data extraction, data from a single, randomized pilot article was independently extracted by one reviewer (author: Neil B. Huben) and checked by a second reviewer (author: Jason M. Johanning).

## 3. Results

### 3.1. Yield

**Figure 1:**
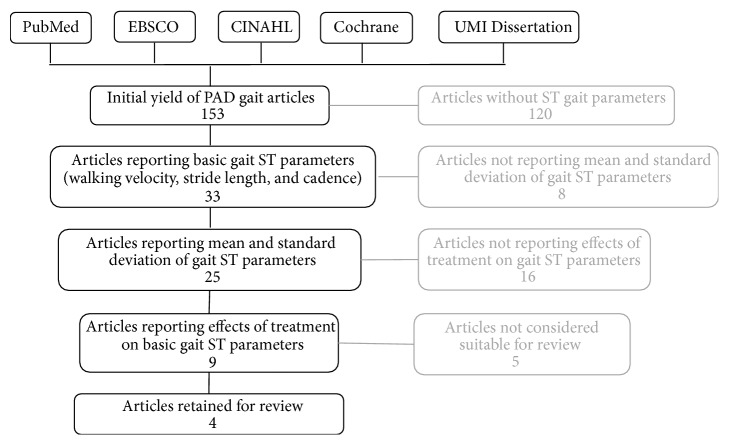
Diagram showing the steps of the article selection procedure which resulted in four articles being selected for detailed review.

The online database search resulted in an initial yield of 153 articles related to PAD and gait (Figure 1). Of those 153 articles, 33 articles reported one or more basic ST gait parameters (velocity, stride length, and cadence; Table 2). Of those 33, 25 articles reported mean and standard deviations of walking velocity, stride length, and cadence in patients with PAD. No articles reported mean step width before and after intervention. Of those 25, 9 examined the effects of treatment on the above ST parameters. After searching the electronic databases and references lists, the final yield included four articles pertaining to the effect of treatments on basic ST gait parameters in patients with PAD. The four articles contained three treatments exploring exercise [36, 38, 39] and one treatment exploring revascularization [37]. None of the articles that reported ST gait parameters before and after pharmacological treatment were considered appropriate for detailed consideration.

### 3.2. Quality of Reviewed Articles

The quality of the four reviewed articles is reported in Tables 2 and 3. The reviewed articles adequately stated the aims, performed a treatment, reported sufficient patient demographics, stated inclusion and exclusion criteria, reported adequate methodology, logically interpreted results, and adequately answered the research questions. Furthermore, the clinical implications were stated in each article.

### 3.3. Demographics

Within the four articles, 66 patients with PAD and 30 controls were enrolled and completed a baseline measurement and at least one posttreatment measurement with respect to the basic gait parameters of interest (e.g., walking velocity, cadence, and stride length). These results are reported in Table 3. All four articles reported pre- and posttreatment walking velocity [36–39]. Two articles reported pre- and posttreatment cadence [36, 37]. Two articles reported pre- and posttreatment stride length [36, 37]. Importantly, no statistically significant differences were reported for the effect of treatments on walking velocity, cadence, or stride length in any of the articles.

## 4. Study Outcomes

All outcomes (ankle-brachial index, walking velocity, cadence, and stride length) of the studies are shown in Table 3. These results are presented based on intervention type: revascularization and exercise. Due to the different interventions, and because outcomes were assessed at a different treatment length for each exercise study, the data could not be pooled. Thus, the results of the individual studies are concisely stated in Sections 4.1 and 4.2.

### 4.1. Revascularization

One study examined functional status before and three-to-four months after infrainguinal bypass in 20 symptomatic patients with Fontaine stage III or IV PAD [37]. Results of the study indicated significant improvements in resting ankle-brachial index; however, no significant changes were reported for walking velocity, cadence, or step length.

### 4.2. Exercise

Three studies evaluated ST gait parameters at baseline and following an exercise intervention in PAD patients. Of those three studies, one included walking velocity, cadence, and stride length as outcome measures [36] and two others included walking velocity only [38, 39]. Regarding length of intervention, one study evaluated patients after 12 months [36], one study after six months [38], and one study after three months [39], respectively. None of the three studies found significant improvements in the outcome measures following intervention.

## 5. Discussion

ST gait parameters provide an overall marker of functional status and health risk in older adults [9, 10] and are associated with disability and hospitalization in older adults [10]. Thus, examining ST gait parameters will provide a vital understanding of the functional status of patients with PAD. From the results of the current systematic literature review, it has been demonstrated that there is lack of studies evaluating ST gait parameters before and after treatment therapies. Specifically, we were unable to find any study that appropriately reported ST gait parameters before and after pharmacological treatment. Further, only one study was found that reported ST gait parameters before and after revascularization. The systematic review of literature in conjunction with the current experimental findings found no significant ST gait parameter changes (walking velocity, cadence, and stride length) due to either exercise or revascularization. Taken as a whole, our current study demonstrates the need for further research to evaluate treatment therapies with a clear and specific focus on ST and more detailed gait parameters. Though limited in nature, the available studies demonstrate that treatments do not change ST gait abnormalities [36–39].

One potential reason behind the failure of revascularization to elicit change could be inability of revascularization to reverse the known myopathic damage in patients with PAD. Patients with symptomatic PAD suffer from lower extremity cellular abnormalities. Such abnormalities include axonal nerve loss and mitochondrial dysfunction [4–6], which could explain the failure to elicit change with revascularization. Lack of differences before and after revascularization could also suggest that even more detailed methods of gait analysis may be necessary to evaluate the effectiveness of currently available treatments. It is possible that ST gait parameters were not sensitive enough to detect the effect of these treatments. Studies of patients with PAD have demonstrated decreased walking velocity, cadence, stride length, and single-support phase and increased double-support phase as compared to controls during pain-free conditions [15, 19]. When patients are asked to walk during claudication pain, further decrements in ST gait parameters are demonstrated [20]. However, two studies that have compared patients with PAD with or without intermittent claudication to controls found no significant differences in ST parameters [2, 45]. These conflicting results have questioned the sensitivity of ST gait parameters to detect differences in gait of patients with PAD. In contrast, advanced biomechanical analysis has revealed changes due to treatment, in previous studies of orthotics, prosthetics, and the elderly, when no change in ST gait parameters has been demonstrated [63, 64]. It may be necessary, therefore, to use advanced biomechanical analysis of joint torques and powers, to confirm whether ST parameters are sensitive enough to evaluate the effectiveness of various treatments used for PAD [18, 65, 66].

Advanced biomechanical analyses allow an objective measurement of the joint muscular responses and contributions to walking during every instance of the gait cycle. Several studies in patients with PAD have included advanced biomechanical gait analysis beyond ST gait parameters, demonstrating abnormal joint kinematics and kinetics [18, 20, 65, 66]. Patients with PAD have significantly reduced ankle plantar-flexor torque and power during late stance with reduced knee power during early and midstance of the gait cycle [65]. These decrements in gait are seen in the unaffected limb of unilateral patients with PAD, demonstrating the impact of this disease on functional status [65]. Thus, the use of advanced biomechanical analysis to assess changes in gait following exercise and surgical treatments must be explored in future studies.

Despite multiple studies documenting the patency rate of treatments in patients with PAD, there was a paucity of articles evaluating ST gait parameters following pharmacological, exercise, or revascularization treatments, severely limiting our ability to perform any meta-analysis. No articles were found for the effect of pharmacological treatment on ST gait parameters that included both pre- and posttreatment measurements using a within-patient design. In addition, only one article was found for the effect of revascularization on ST gait parameters, which focused solely on lower extremity bypass. Potential reasons for the limited data include publication bias and lack of publications due to negative study results. We attempted to limit this effect by searching multiple databases, including dissertation work. A formal meta-analysis, which aims to quantitatively combine the results of different studies, could not be conducted in the current study because the four articles selected for inclusion differed in terms of the type of treatment and/or the length of time for the treatment. Although walking velocity was assessed prior to and following the treatment in each of the four articles, three of the articles dealt with an exercise treatment, but the exercise programs were of different durations: 3 months, 6 months, and 12 months, respectively [36, 38, 39]. The fourth article assessed ST gait parameters three to four months after revascularization [37]. Based on the yield of articles, additional studies that extend to 6 months or longer follow-up are needed for both exercise and surgical treatments.

In conclusion, our study demonstrates significant lack of research examining the effectiveness of treatments on ST gait parameters in patients with PAD. From the four articles found which reported pre- and posttreatment data, no significant changes were demonstrated due to exercise or revascularization. Due to the limited data, additional studies are needed to confirm these results. Further studies need to be performed to adequately address in a detailed and scientific manner the effects of revascularization or alternative exercise protocols on spatial and temporal gait parameters, in addition to accepted clinical measurements, to provide a comprehensive understanding of how such protocols may alter the gait of patients with PAD. Moreover, advanced biomechanical gait analysis should be performed to provide objective evaluation of intrinsic components to gait (i.e., joint torques and powers) to confirm the sensitivity of ST gait parameters to functional changes in patients with PAD following intervention.

## Figures and Tables

**Table 1:** Summary of quality assessment for individual articles. The four articles shown are those selected for detailed review. Scoring criteria employed: Yes, 1; inadequate, 0.5; no, 0.

Question	Crowther et al. [36]	Gardner and Killewich [37]	Wullink et al. [38]	McDermott et al. [39]
(1) Research aims stated	1	1	1	1
(2) Intervention clearly stated	1	1	1	1
(3) Patient demographics reported				
Number	1	1	1	1
Age	1	1	1	1
Sex	1	1	1	1
Height	1	0	0	0
BMI	1	0	1	1
ABI	1	1	1	1
(4) Methods: recruitment explained	1	1	0.5	1
(5) Methods: inclusion and exclusion criteria reported	0.75	0.5	1	1
(6) Methods: control group	1	0	1	0
(7) Methods: adequate methodology reported	1	1	1	1
(8) Methods: methodology able to answer research question				
Sample size	1	1	1	1
Equipment	1	1	1	1
Intervention	1	1	1	1
Data processing	1	1	1	1
Statistical analysis	1	1	1	1
(9) Methods: reliability of methodology	1	1	1	1
(10) Results: logical interpretation of data	1	1	1	1
(11) Results: reported key findings supported by data	1	1	1	1
(12) Discussion: research question answered adequately	1	1	1	1
(13) Discussion: clinical implications stated	1	1	1	1

Total	21.75	18.50	20.50	20.00

**Table 2:** List of articles resulting from a search of literature databases (PubMed, EBSCO, CINAHL, Cochrane library, and UMI Dissertation). Only the articles containing basic ST gait parameters are listed. Articles shown in bold print contained information pertaining to the effect of treatment on basic ST gait parameters in patients with PAD, which met the criteria for detailed review.

Ref. number	Reference	Title	Treatment	Gait parameters	Mean ± SD
[40]	Arseven et al. (2007)	Does Lower-Extremity Arterial Disease Predict Future Falling among Older Men and Women?	None	Velocity	Y

[18]	Chen et al. (2008)	Bilateral Intermittent Claudication Results in Alterations in the Gait Biomechanics at the Hip and Ankle Joints during Gait	None	Velocity, cadence, and stride length	Y

[20]	Crowther et al. (2007)	Relationship between Temporal-Spatial Gait Parameters, Gait Kinematics, Walking Performance, Exercise Capacity, and Physical Activity Level in Peripheral Arterial Disease	None	Velocity, cadence, and stride length	Y

**[36]**	**Crowther et al. (2008)**	**Effects of a Long-Term Exercise Program on Lower Limb Mobility, Physiological Responses, Walking Performance, and Physical Activity Levels in Patients with Peripheral Arterial Disease**	**Exercise program**	**Velocity, cadence, and stride length**	**Y**

[41]	Dolan et al. (2002)	Peripheral Artery Disease, Diabetes, and Reduced Lower Extremity Functioning	None	Velocity	N

[19]	Gardner et al. (2001)	Altered Gait Profile in Subjects with Peripheral Arterial Disease	None	Velocity, cadence, and stride length	Y

[42]	Gardner et al. (2004)	Natural History of Physical Function in Older Men with Intermittent Claudication	Time	Velocity	Y

**[37]**	**Gardner and Killewich (2001)**	**Lack of Functional Benefits following Infrainguinal Bypass in Peripheral Arterial Occlusive Disease Patients**	**Bypass**	**Velocity, cadence, and stride length**	**Y**

[43]	Giri et al. (2006)	Statin Use and Functional Decline in Patients with and without Peripheral Arterial Disease	Statin	Velocity	Y

[44]	Kuo and Yu (2008)	The Relation of Peripheral Arterial Disease to Leg Force, Gait Speed, and Functional Dependence among Older Adults	None	Velocity	Y

[45]	McCully et al. (1999)	The Effects of Peripheral Vascular Disease on Gait	Treadmill (immediate)	Velocity, stride length	Y

[46]	McDermott et al. (2000)	Asymptomatic Peripheral Arterial Disease Is Independently Associated with Impaired Lower Extremity Functioning: The Women's Health and Aging Study	None	Velocity	Y

[2]	McDermott et al. (2001)	Gait Alterations Associated with Walking Impairment in People with Peripheral Arterial Disease with and without Intermittent Claudication	None	Velocity, cadence, and stride length	N

[47]	McDermott et al. (2001)	Leg Symptoms in Peripheral Arterial Disease: Associated Clinical Characteristics and Functional Impairment	None	Velocity	Y

[48]	McDermott et al. (2004)	Functional Decline in Peripheral Arterial Disease: Associations with the Ankle Brachial Index and Leg Symptoms	None	Velocity	Y

[17]	McDermott et al. (2008)	Asymptomatic Peripheral Arterial Disease Is Associated with More Adverse Lower Extremity Characteristics Than Intermittent Claudication	None	Velocity	N

[49]	McDermott et al. (2003)	Statin Use and Leg Functioning in Patients with and without Lower-Extremity Peripheral Arterial Disease	Statin	Velocity	Y

[50]	McDermott et al. (2003)	Depressive Symptoms and Lower Extremity Functioning in Men and Women with Peripheral Arterial Disease	None	Velocity	N

[51]	McDermott et al. (2005)	Functional Decline in Lower-Extremity Peripheral Arterial Disease: Associations with Comorbidity, Gender, and Race	None	Velocity	Y

[52]	McDermott et al. (2006)	Obesity, Weight Change, and Functional Decline in Peripheral Arterial Disease	None	Velocity	N

[53]	McDermott et al. (1998)	Measurement of Walking Endurance and Walking Velocity with Questionnaire: Validation of the Walking Impairment Questionnaire in Men and Women with Peripheral Arterial Disease	None	Velocity	Y

[54]	McDermott et al. (2007)	Baseline Functional Performance Predicts the Rate of Mobility Loss in Persons with Peripheral Arterial Disease	None	Velocity	Y

[55]	McDermott et al. (2007)	Physical Activity, Walking Exercise, and Calf Skeletal Muscle Characteristics in Patients with Peripheral Arterial Disease	None	Velocity	Y

[56]	McDermott et al. (2004)	Leg Strength in Peripheral Arterial Disease: Associations with Disease Severity and Lower-Extremity Performance	None	Velocity	N

**[57]**	**McDermott et al. (2006)**	Functional Decline in Patients with and without Peripheral Arterial Disease: Predictive Value of Annual Changes in Levels of C-Reactive Protein and D-Dimer	**Time**	Velocity	Y

[58]	McDermott et al. (2004)	Inflammatory and Thrombotic Blood Markers and Walking-Related Disability in Men and Women with and without Peripheral Arterial Disease	None	Velocity	Y

[59]	McDermott et al. (2003)	Sex Differences in Peripheral Arterial Disease: Leg Symptoms and Physical Functioning	None	Velocity	N

**[39]**	**McDermott et al. (2004)**	**A Pilot Exercise Intervention to Improve Lower Extremity Functioning in Peripheral Arterial Disease Unaccompanied by Intermittent Claudication**	**Exercise program**	**Velocity**	**Y**

[60]	McDermott et al. (2007)	Lower Extremity Ischemia, Calf Skeletal Muscle Characteristics, and Functional Impairment in Peripheral Arterial Disease	None	Velocity	Y

[61]	Rucker-Whitaker et al. (2004)	Peripheral Arterial Disease in African Americans: Clinical Characteristics, Leg Symptoms, and Lower Extremity Functioning	None	Velocity	N

[15]	Scherer et al. (1998)	Gait Characteristics of Patients with Claudication	None	Velocity, cadence, and stride length	Y

[62]	Scherer et al. (2006)	Lack of Relationship between Gait Parameters and Physical Function in Peripheral Arterial Disease	None	Velocity, cadence, and stride length	Y

**[38]**	**Wullink et al. (2001)**	**A Primary Care Walking Exercise Program for Patients with Intermittent Claudication**	**Walking exercise**	**Velocity**	**CI**

**Table 3:** Study information and parameters reported for the four articles that contained information pertaining to the effect of treatment on basic ST gait parameters in patients with PAD, which were selected for detailed analysis.

Study	Crowther et al. [36]	McDermott et al. [39]	Wullink et al. [38]	Gardner and Killewich [37]
Number of subjects	10	24	17	20
Number of controls	11	—	8	—
Intervention	Exercise	Exercise	Exercise	Bypass
Time (months)	12	6	3	3-4

Parameters reported^*∗*^				
Ankle-brachial index	P	P	P^**#**^	P
Walking velocity	P	P	P	P
Cadence	P	—	—	P
Stride length	P	—	—	P

^*∗*^Mean and standard deviation of the listed parameters were reported.

^#^Postintervention measurements were not reported.
